# Modeling the Evolution of Dynamic Triadic Closure Under Superlinear Growth and Node Aging in Citation Networks

**DOI:** 10.3390/e27090915

**Published:** 2025-08-29

**Authors:** Li Liang, Hao Liu, Shi-Cai Gong

**Affiliations:** School of Sciences, Zhejiang University of Science and Technology, Hangzhou 310023, China; hitliangli@163.com (L.L.); 1319919617abc@gmail.com (H.L.)

**Keywords:** citation networks, network evolution, triangular structure, superlinear growth, node aging

## Abstract

Citation networks are fundamental for analyzing the mechanisms and patterns of knowledge creation and dissemination. While most studies focus on pairwise attachment between papers, they often overlook compound relational structures, such as co-citation. Combining two key empirical features, superlinear node inflow and the temporal decay of node influence, we propose the Triangular Evolutionary Model of Superlinear Growth and Aging (TEM-SGA). The fitting results demonstrate that the TEM-SGA reproduces key structural properties of real citation networks, including degree distributions, generalized degree distributions, and average clustering coefficients. Further structural analyses reveal that the impact of aging varies with structural scale and depends on the interplay between aging and growth, one manifestation of which is that, as growth accelerates, it increasingly offsets aging-related disruptions. This motivates a degenerate model, the Triangular Evolutionary Model of Superlinear Growth (TEM-SG), which excludes aging. A theoretical analysis shows that its degree and generalized degree distributions follow a power law. By modeling interactions among triadic closure, dynamic expansion, and aging, this study offers insights into citation network evolution and strengthens its theoretical foundation.

## 1. Introduction

Among the various types of networks, citation networks have long served as a cornerstone of scholarly inquiry, providing a quantitative framework for examining the mechanisms behind knowledge creation and dissemination [1,2]. Analyzing the evolutionary patterns of citation networks helps researchers to identify emerging research directions and assists policymakers in formulating effective strategies, thereby underscoring the broader significance of understanding network evolution [3].

Early models of citation network evolution typically assumed linear growth, with one node or edge added at each time step [4,5]. However, empirical studies have shown that real-world citation networks often exhibit accelerated growth, leading to models with time-dependent growth rates [6,7,8,9]. Dorogovtsev and Mendes [10] formalized this concept, showing that the number of new links scales as a power law $t^{\alpha }$ with α>1. Independently, Smith et al. [11] introduced a differential framework to characterize network acceleration, defining it as the time derivative of the ratio between the rates of link and node additions. Beyond edge acceleration, researchers have also examined superlinear node addition [12,13,14]. For example, Jung et al. [15] proposed that each node generates new nodes in proportion to its degree, resulting in exponential network growth. In a different vein, Li et al. [16] introduced a flexible hub attraction mechanism, where m·k(t−1) new nodes are added per time step, and Song et al. [17] defined a multiplicative growth process, which leads to self-similar network structures. Additionally, Liu et al. [18] incorporated $t^{\theta }$ node insertions per step to capture bursty patterns of scholarly activity. Collectively, these models better reflect the fast-evolving dynamics of citation networks, particularly the rapid emergence of high-degree nodes or “supernodes” within short timescales.

In addition to accelerated growth, citation networks exhibit signs of decline. Specifically, newly published papers tend to cite more recent influential works, while older papers are increasingly neglected—a phenomenon known as node aging [19,20,21,22]. To model this temporal decay of influence, Hu et al. [23] proposed a power-law decay function $\tau^{\alpha }$, where τ denotes node age and α is the decay exponent. Although such traditional citation-aging models effectively characterize the temporal decline in citation frequency, some studies indicate that scientific influence can persist for a long time. For instance, Wang et al. [24] developed a mechanistic model combining preferential attachment, aging, and fitness to predict long-term citation dynamics. Separately, Meng et al. [25] introduced the concept of “hidden citations,” referring to works that continue to influence academic discourse despite not being explicitly cited. Taken together, these perspectives emphasize the need for modeling frameworks that capture both the visible and latent dimensions of scientific impact.

Despite advances in evolutionary models that enhance our understanding of citation networks, most existing approaches rely on pairwise attachment mechanisms [26]. However, real-world citation behavior often involves triangle-based or transitive interaction patterns. For instance, authors tend to cite not only influential papers but also works that are co-cited with them or that cite them, thereby forming clustered and transitive citation motifs. To address this limitation, we incorporate triadic closure into the citation network growth process, aiming to better reproduce the triangle-based citation patterns observed in empirical data.

Triadic closure is often considered an important mechanism contributing to the emergence of clustered and community structures in real-world networks. Davidsen et al. [27] introduced an acquaintance-based model that generates high clustering and short paths through local triangle formation—a feature later incorporated into preferential attachment models by Holme et al. [28] to enable tunable clustering in scale-free networks. Bianconi et al. [29] highlighted the role of triadic closure in promoting community formation, while Kunegis et al. [30] demonstrated that transitive closure alone can give rise to power-law degree distributions. Other analytical frameworks have sought to probabilistically close open triads or guide their closure based on structural proximity [31,32]. As a complementary perspective, Cirigliano [33] proposed a static model that achieves exact clustering predictions by closing triads with fixed probabilities on a predefined backbone.

While triangle-based mechanisms have proven effective in achieving clustering, transitivity, and community structure in generic network models, they often fail to account for key temporal dynamics when applied to real-world citation networks, particularly superlinear growth and node aging, which are central to their developmental patterns. To bridge this gap, we propose a triangular growth model that integrates both mechanisms into the citation network evolution process. Our model simultaneously captures the structural expansion of the network and the temporal decay of node influence. By analyzing its emergent properties and comparing them with empirical citation data, we aim to provide a more comprehensive explanation for the emergence and persistence of triangle-rich structures in evolving scholarly networks.

This paper is organized as follows. Section 1 introduces the background, reviews related citation network evolution models, explains the motivation for proposing the TEM-SGA framework, and presents in Table 1 the parameters employed in this study together with their descriptions. Section 2 presents the dataset and details the construction of the proposed model. Section 3 investigates the influence of model parameters on the generated network topology and also presents the simulation of the real citation network. Section 4 introduces the degenerate model TEM-SG derived from the TEM-SGA framework, and provides a theoretical analysis of the TEM-SG model together with a comparison between the analytical results and simulations. Finally, Section 5 summarizes the main findings, discusses the limitations of the study, and outlines directions for future research.

## 2. Materials and Methods

### 2.1. Data Description

We use the DBLP-Citation-network V12 dataset (hosted on Kaggle), which contains 4,894,081 scientific papers and 45,564,149 citation links, along with complete metadata, including publication year and reference lists. This dataset supports our modeling assumptions in two crucial ways. First, the availability of full reference lists allows us to reconstruct triadic closures based on citation and co-citation, which is essential for evaluating our triangle-based attachment model. Second, the longitudinal range and time-stamped entries make it suitable for verifying the superlinear growth and aging hypotheses assumed in our model design. Additionally, DBLP focuses on computer science publications, ensuring a coherent disciplinary scope that reduces heterogeneity introduced by cross-domain citations. This field consistency is beneficial for studying structure-dependent mechanisms such as clustering and degree heterogeneity.

We also evaluated alternative datasets. OpenAlex offers broad and frequently updated coverage, which is advantageous in many contexts, although its evolving nature may challenge strict reproducibility. Cora and similar citation benchmarks are widely used in graph learning, but their small scale—typically a few thousand nodes—makes them unsuitable for studying higher-order phenomena like heavy-tailed triangle participation. Overall, DBLP strikes a good balance between disciplinary coherence, temporal depth, citation completeness, and public accessibility, making it well-suited for our purposes.

**Figure 1:**
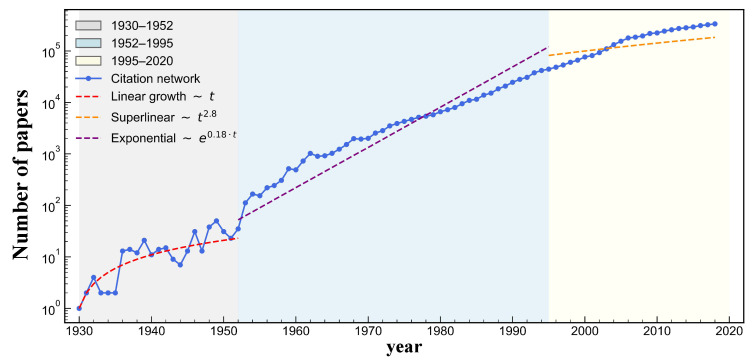
Annual publication volume in the DBLP V12 dataset compared with baselines: linear y=t; superlinear $y=t^{\theta }$ with θ=2.8; exponential $y=\exp\left(\alpha t\right)$ with α=0.18. The blue curve shows the yearly number of papers recorded in the DBLP citation network from 1930 to 2020. The red, orange, and purple curves represent baselines for comparison.

We analyze the annual publication volume in the DBLP V12 citation network over the period 1930–2020, as shown in Figure 1, and compare the empirical curve with three representative baselines: linear, exponential, and superlinear. To better characterize the evolution of publication growth over time, we divide the timeline into three segments—1930–1952, 1952–1995, and 1995–2020—and fit each baseline separately within these intervals. The fitting errors (log–MSE) and coefficients of ($R^{2}$) determination are reported in Table 2.

In the first two stages (1930–1952 and 1952–1995), although linear and exponential growth models provide better fits, the actual scale of the citation network remains relatively limited, with publication volumes still in a steady or exponential expansion phase. In contrast, the period from 1995 to 2020 exhibits a different dynamic pattern. During this stage, the superlinear baseline achieves the lowest log–MSE (0.038), significantly outperforming both the linear and exponential baselines. This indicates that, since 1995, the citation network has entered a phase of superlinear growth in recent decades. One possible reason for this shift is the rise of digital publishing infrastructures and bibliographic platforms (e.g., ScienceDirect and SpringerLink) in the mid-to-late 1990s, which have facilitated broader access to related literature and potentially change citation dynamics.

**Figure 2:**
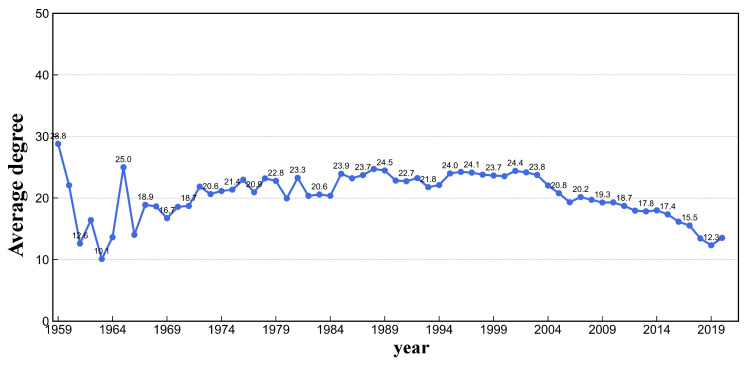
The average degree per year from 1959 to 2020 in the DBLP V12 dataset. Each point represents the average number of citation links associated with papers published in a given year.

To further explore the network’s temporal structural properties, we analyze the average degree per year from 1959 to 2020, as shown in Figure 2. Each point represents the average number of citation links associated with papers published in a given year. The curve shows that the average degree remained relatively stable, fluctuating between 10 and 30.

### 2.2. Model

**Triangular Evolutionary Model of Superlinear Growth and Aging (TEM-SGA).** We propose the TEM-SGA, a triangle-based evolutionary framework that integrates two key mechanisms observed in real-world citation systems: superlinear network growth and node aging.

**Superlinear growth.** Empirical evidence indicates that the citation network has entered a superlinear growth regime since the mid-1990s. To capture this accelerated expansion phase, our model introduces $t^{\theta }$ new nodes at each discrete time step *t* (t=0,1,2,…,n), where θ>1 governs the growth rate. The superlinear formulation reflects the cumulative advantage process observed in recent decades, during which the number of scientific publications increases faster than linearly over time.

**Triadic closure through edge-based attachment.** In real citation behavior, authors not only cite high-impact papers but also reference works that are frequently co-cited with—or that themselves cite—those high-impact papers. This mechanism naturally gives rise to triadic closure. To model this, each new node in the TEM-SGA selects *m* existing edges according to a priority rule and connects to both endpoints of each selected edge, thereby forming one new triangle per chosen edge. Here, nodes represent papers, and edges denote citation relations.

Unlike standard preferential attachment (PA), where nodes are chosen in proportion to their degrees, the TEM-SGA assumes that edges are chosen in proportion to their triangle participation. Specifically, the probability of selecting an edge depends on its triangle count at the previous time step:

(1) $$P_{ij}\left(t\right)=\frac{k_{ij}\left(t-1\right)}{\sum_{\left(u,v)\in E(t-1\right)}k_{uv}\left(t-1\right)},$$

where $k_{ij}\left(t-1\right)$ is the number of triangles involving edge (i,j) at time t−1. This rule ensures that edges embedded in dense local structures are more likely to attract further connections.

**Aging effect.** Over time, older nodes gradually lose their attractiveness to new arrivals as authors tend to cite more recent literature. We capture this through an exponential decay in node attractiveness:

(2) $$a_{i}=k_{i}e^{-\lambda (t-t_{i})},$$

where $a_{i}$ is the attractiveness of node *i*, $k_{i}$ is the degree of node *i*, $t_{i}$ is its birth time, and λ controls the aging rate [34]. A larger λ produces faster decay, meaning that old nodes are cited less frequently.

Combining Equations (1) and (2), the final probability of selecting edge (i,j) at time *t* becomes

(3) $$P_{ij}\left(t\right)=\frac{\left(\frac{k_{i}e^{-\lambda (t-t_{i})}+k_{j}e^{-\lambda (t-t_{j})}}{k_{i}+k_{j}}\right)k_{ij}\left(t-1\right)}{\sum_{\left(u,v)\in E(t-1\right)}\left(\frac{k_{u}e^{-\lambda (t-t_{u})}+k_{v}e^{-\lambda (t-t_{v})}}{k_{u}+k_{v}}\right)k_{uv}\left(t-1\right)}.$$

This formulation integrates both structural reinforcement (via triangle counts) and temporal decay (via node aging). Edges incident to older nodes receive smaller weights due to the exponential term, thereby lowering their selection probability.

**Model implementation.** The TEM-SGA procedure can be summarized as follows:

- Initial network: The process begins with a fully connected network of $N_{0}\left(N_{0}\geq 3\right)$ nodes, ensuring that each edge is initially part of a triangle.
- Growth mechanism: At each discrete time step *t*, the network grows by introducing $t^{\theta }$ new nodes. Each new node selects *m* existing edges according to Equation (3), under the constraint that the selected edges must have pairwise-disjoint endpoints. The new node then connects to both endpoints of each chosen edge, thereby forming exactly *m* new triangles. This rule enforces structural consistency and prevents the creation of multi-edges.
- Fallback rule: If the network at time *t* contains fewer than *m* usable edges, it is impossible for each new node to attach to *m* disjoint edges. In this case, a fallback mechanism is applied: each new node instead attaches randomly to exactly one existing node. Under this mode, $t^{\theta }$ new edges are added but no new triangles are formed. This ensures continuous network expansion even under structural constraints.
- Termination: The process stops once the total number of nodes reaches the predefined size *N*.

**Figure 3:**
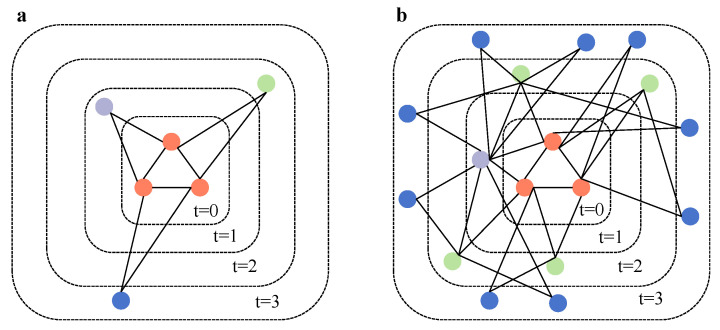
Schematic diagram of (**a**) the evolution process of BA model and (**b**) the TEM-SGA under parameter setting (m=1, θ=2, λ=0.5).

In summary, the TEM-SGA integrates superlinear growth, triadic closure, and node aging into a unified generative model. The combination of these mechanisms allows the model to replicate both the structural heterogeneity and the temporal dynamics observed in empirical citation networks (see Figure 3 and Algorithm 1).
**Algorithm 1:** TEM-SGA: Triangular Evolutionary Model of Superlinear Growth and Aging
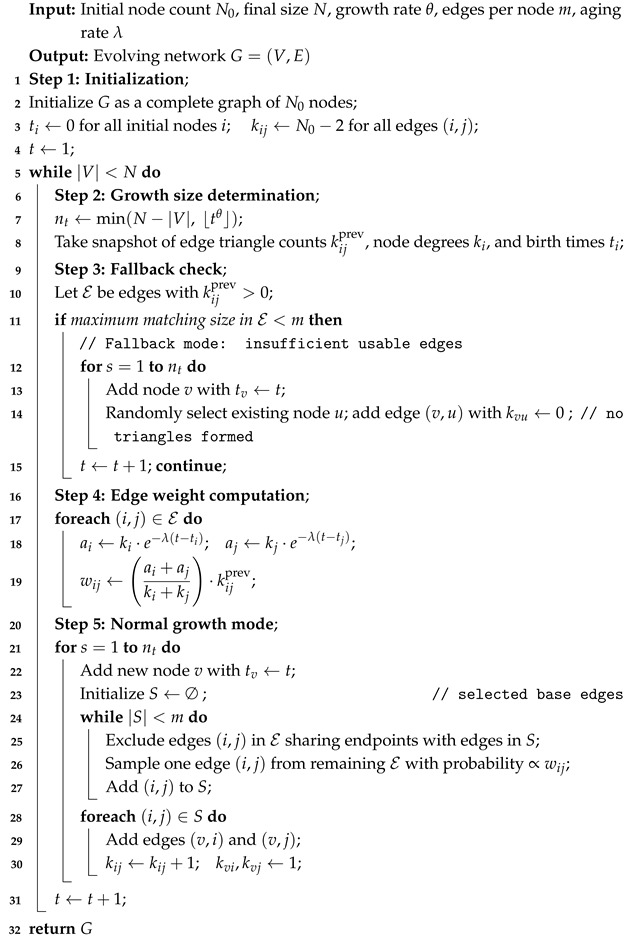


## 3. Results and Discussion

### 3.1. Network Topology

In this section, we conduct a series of experiments to examine how the combined effects of growth rate and node aging influence the structural properties of networks generated by the TEM-SGA.

**Figure 4:**
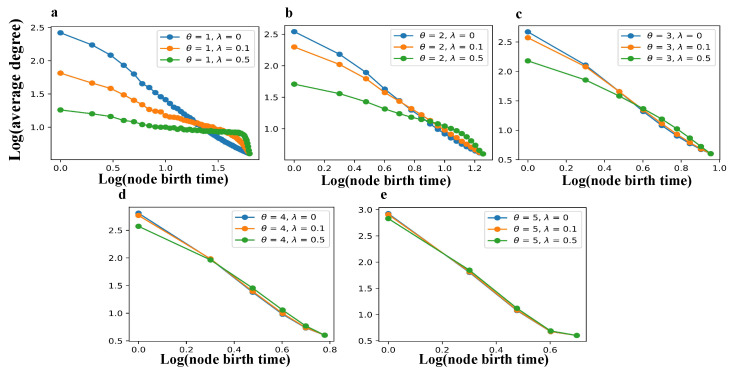
Birth time determines average degree in the TEM-SGA (log–log plots). The average degree is shown as a function of node birth time under different growth exponents θ and aging coefficients λ with m=2 fixed. (**a**) θ=1, λ∈{0,0.1,0.5}; (**b**) θ=2, λ∈{0,0.1,0.5}; (**c**) θ=3, λ∈{0,0.1,0.5}; (**d**) θ=4, λ∈{0,0.1,0.5}; (**e**) θ=5, λ∈{0,0.1,0.5}. Each panel reports simulations on networks with N=2000 nodes, averaged over 100 realizations.

**Figure 5:**
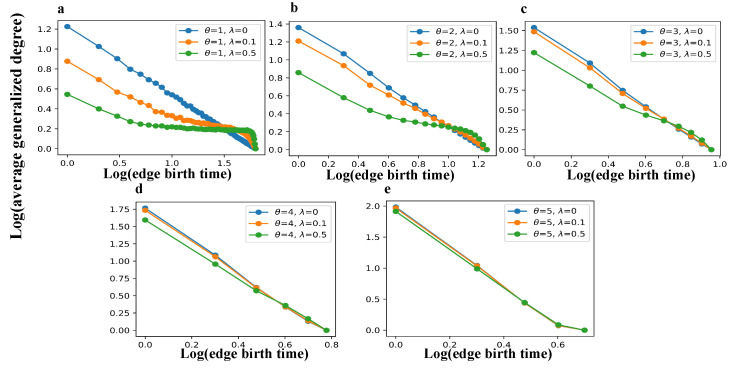
Birth time determines average generalized degree in the TEM-SGA (log–log plots). The average generalized degree is shown as a function of edge birth time under different growth exponents θ and aging coefficients λ with m=2 fixed. (**a**) θ=1, λ∈{0,0.1,0.5}; (**b**) θ=2, λ∈{0,0.1,0.5}; (**c**) θ=3, λ∈{0,0.1,0.5}; (**d**) θ=4, λ∈{0,0.1,0.5}; (**e**) θ=5, λ∈{0,0.1,0.5}. Each panel reports simulations on networks with N=2000 nodes, averaged over 100 realizations.

Figure 4 and Figure 5 jointly examine how birth time influences structural advantages at both the node and edge levels within the TEM-SGA. Specifically, Figure 4 tracks the evolution of the average node degree over node joining time, whereas Figure 5 examines the average generalized degree (average edge triangle count) with respect to edge birth time.

A consistent pattern emerges across both figures: as the aging coefficient λ increases, the structural advantages of early-born nodes and edges—as reflected in their higher average degrees and generalized degrees—decline substantially. This demonstrates that aging effectively suppresses the cumulative advantage mechanism by constraining the long-term growth potential of early network elements. Such behavior aligns with the findings of Dorogovtsev and Mendes [35], who showed that, under aging dynamics (α>0), early nodes gradually lose their dominance in evolving network structures. Meanwhile, as the growth exponent θ increases, the network grows rapidly, causing many new nodes to join before older nodes accumulate substantial structural advantages. As a result, the degree and generalized degree disparities associated with birth time are reduced, and the effects of aging become less pronounced. Collectively, these results suggest that, while aging attenuates the structural accumulation linked to birth order, superlinear expansion mitigates this effect by reshaping the temporal dynamics of network expansion.

Although both node-level and edge-level structures are shaped by the interplay of growth (θ) and aging (λ), their sensitivities differ. For average node degree (Figure 4b–d), increasing θ leads to stronger convergence across different λ values compared to average edge generalized degree (Figure 5b–d), indicating that faster network expansion more effectively offsets aging at the node level than at the edge level. This comparison reveals an asymmetry in structural dynamics: superlinear growth exerts a stronger moderating effect on node connectivity, whereas aging has a more persistent impact on edge-level closure.

**Figure 6:**
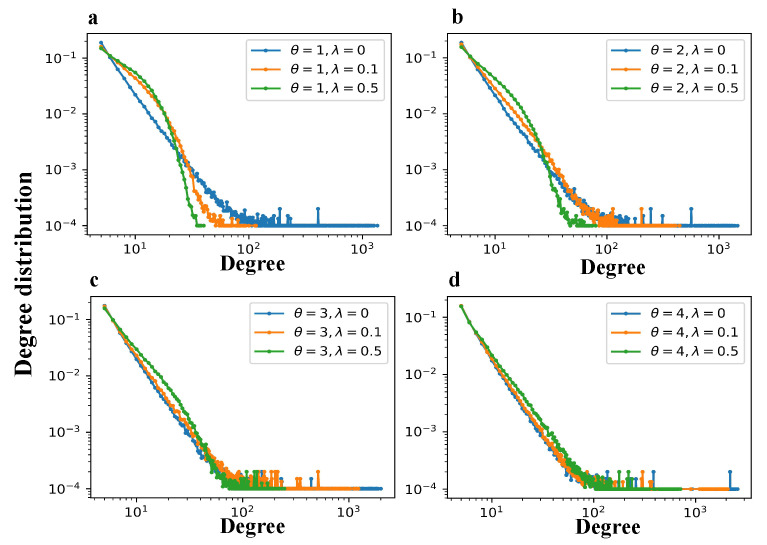
Degree distributions under varying aging and growth parameters in the TEM-SGA (log–log plots). The degree distribution P(k) is shown for networks generated with fixed m=2 under different growth exponents θ and aging coefficients λ. (**a**) θ=1, λ∈{0,0.1,0.5}; (**b**) θ=2, λ∈{0,0.1,0.5}; (**c**) θ=3, λ∈{0,0.1,0.5}; (**d**) θ=4, λ∈{0,0.1,0.5}. Each panel reports results averaged over 100 realizations for networks with N=10,000 nodes.

**Figure 7:**
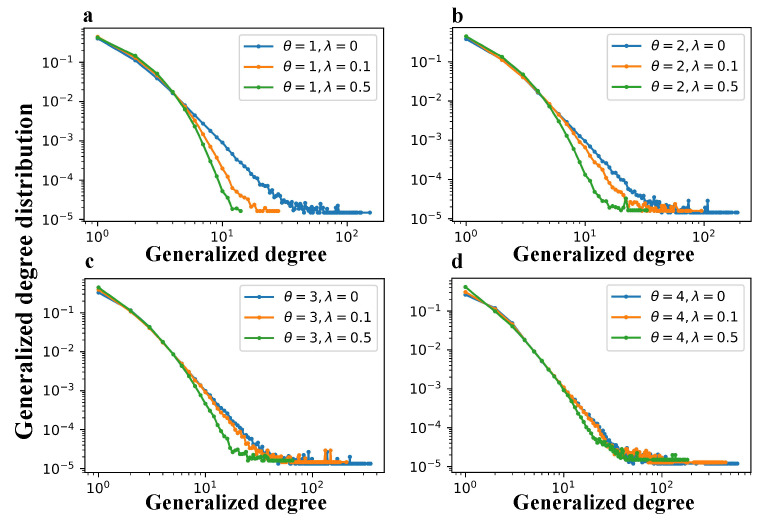
Generalized degree distributions under varying aging and growth parameters in the TEM-SGA (log–log plots). The generalized degree distribution $P(k_{\ell })$ is shown for networks generated with fixed m=2 under different growth exponents θ and aging coefficients λ. (**a**) θ=1, λ∈{0,0.1,0.5}; (**b**) θ=2, λ∈{0,0.1,0.5}; (**c**) θ=3, λ∈{0,0.1,0.5}; (**d**) θ=4, λ∈{0,0.1,0.5}. Each panel reports results averaged over 100 realizations for networks with N=10,000 nodes.

Figure 6 and Figure 7 show the degree distributions and generalized degree distributions, respectively, for networks generated by the TEM-SGA under different combinations of the growth exponent θ and aging coefficient λ.

Across all the subfigures (a–d), increasing the aging coefficient λ significantly reduces both the maximum node degree and the maximum generalized degree. This result demonstrates that aging suppresses hub formation by reducing the long-term attractiveness of early nodes and edges, thereby weakening preferential attachment over time. Closer inspection further reveals that aging impacts the low-to-medium degree regions of the two distributions in distinct ways. In the degree distribution P(k), increasing λ substantially increases the probability mass within the range k∈[5,30]. In contrast, for the generalized degree distribution $P(k_{\ell })$, the most pronounced effect occurs at very small values, where there is a slight increase in the probability of links with $k_{\ell }\in \left[1,3\right]$.

Taken together, these local distortions in both the degree and generalized degree distributions reflect a deeper structural shift. Specifically, the introduction of aging indeed disrupts the emergence of clear power-law distributions. Furthermore, distributions under higher λ values deviate more markedly from the ideal power-law form. However, as θ increases, the divergence among curves corresponding to different λ values becomes less pronounced. This convergence suggests that superlinear growth mitigates the structural disruptions caused by aging: as more nodes are added at each time step with increasing θ, the relative influence of early network elements diminishes more slowly, thereby reducing the effectiveness of aging in shaping heterogeneity. These results indicate that aging-induced disruption of power-law behavior is not absolute but instead depends on the relative balance between θ and λ. It should be noticed that both Figure 6 and Figure 7 include occasional extreme values as the results are averaged over 100 independent simulation runs, during which such outliers are inevitable.

### 3.2. Empirical Validation of Structural Features in Citation Networks

For subsequent analyses that require detailed structural simulations, we selected a representative subset of the DBLP-Citation-network V12 dataset, namely DBLP [36], a well-established dataset in complex network and scientometric studies. This dataset contains 12,591 nodes and 49,743 edges and has been widely used to investigate power-law distributions, preferential-attachment mechanisms, and network-evolution models.

**Figure 8:**
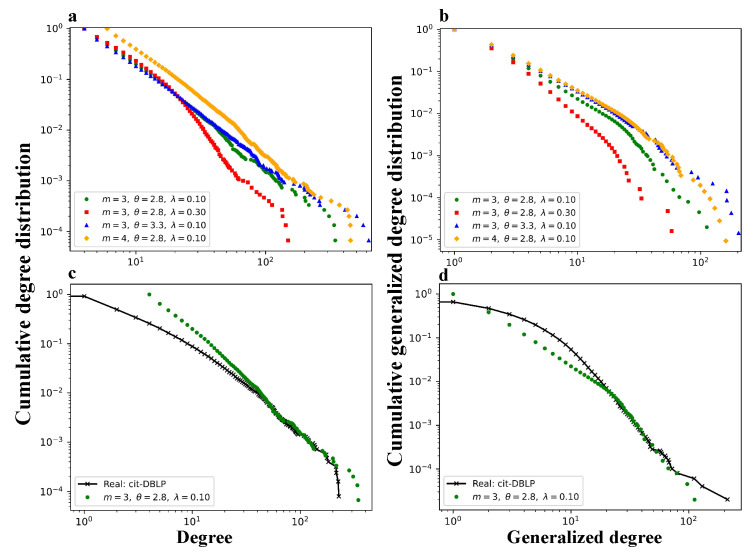
Comparison of cumulative degree and generalized degree distributions, including real-world data from the DBLP citation network. Subfigures (**a**,**b**) show the cumulative degree distributions and cumulative generalized degree distributions, respectively, generated by the TEM-SGA under various parameter settings (*m*, θ, λ), with the number of nodes fixed at N=15,000. Subfigures (**c**,**d**) compare the model-generated results (in green) with empirical distributions from the DBLP citation network (in black) using the best-fitting parameter combination (m=3, θ=2.8, λ=0.10).

Figure 8 presents a comprehensive comparison of the cumulative degree and generalized degree distributions generated by the TEM-SGA under various parameter settings, alongside those observed in the real DBLP citation network.

Figure 8a illustrates that the cumulative degree distribution responds systematically to changes in model parameters. Increasing *m* (green vs. yellow curves) consistently shifts the entire distribution upward, indicating that allowing each new node to select more edges raises the overall degree level in the network. Raising θ (green vs. blue curves) concentrates more probability mass in the intermediate degree range (k∈[40,200]), extending the distribution tail and increasing the maximum degree. This suggests that a higher θ expedites node inflow, intensifies early preferential attachment, and enables a small number of nodes to accumulate high degrees. In contrast, increasing λ (green vs. red curves) strongly suppresses the distribution tail as aging penalizes older nodes, limiting their long-term link accumulation and effectively capping the maximum degree.

Figure 8b reveals distinct patterns in the cumulative generalized degree distributions. Increasing *m* (green vs. yellow curves) slightly elevates the overall distribution curve. Compared to its more substantial impact on the cumulative degree distribution in Figure 8a, this uplift is relatively weaker. Additionally, increasing θ (green vs. blue curves) results in a global elevation of the distribution, with the uplift being notably steeper in the mid-to-high range ($k_{\ell }\geq 30$) than in the lower range ($0\leq k_{\ell }\leq 30$). In contrast, raising λ (green vs. red curves) causes a marked downward shift across the entire distribution, especially reducing the probability of generalized degrees exceeding $k_{\ell }\approx 3$. This pattern is consistent with that observed in Figure 7, indicating that a stronger aging effect significantly suppresses the likelihood of edges participating in triangle formation, thereby weakening structural clustering within the network.

Figure 8c,d compare the TEM-SGA to empirical data from the DBLP network using the parameter set (m=3, θ=2.8, λ=0.1), which provides the closest visual fit. Despite minor discrepancies in the low-degree regime ($k,k_{\ell }≲20$), the model reproduces the upper tail behavior well, accurately capturing the distributional trends of both degree and generalized degree. These results suggest that, under appropriate parameter configurations, the TEM-SGA can replicate, to some extent, the structural features observed in real-world citation networks.

As shown in Table 3, classical models such as BA, HK, KE-D, and LC can produce networks with measurable clustering coefficients. That said, the resulting average clustering coefficients (ACCs) still differ considerably from those measured in the DBLP citation network. By contrast, the proposed TEM-SGA is designed to integrate co-citation mechanisms more directly into the generative rules, thereby yielding clustering levels that more closely resemble empirical observations and simultaneously capturing key structural features such as degree distributions and generalized degree distributions. Moreover, the model parameters (*m*, θ, and λ) allow flexible and precise adjustment of the clustering level.

## 4. Degenerate Model

**Figure 9:**
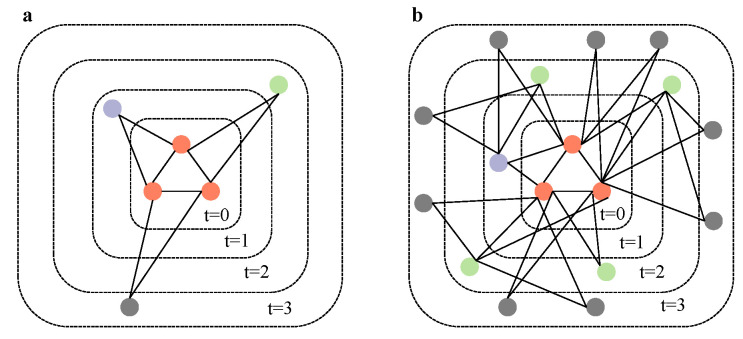
Schematic diagram of (**a**) the evolution process of BA model and (**b**) the TEM-SG under parameter setting (m=1, θ=2).

**Triangular Evolutionary Model of Superlinear Growth (TEM-SG).** Our previous results suggest that, when the growth effect is sufficiently strong relative to aging, it can offset the structural disparities introduced by aging. Motivated by this observation, we introduce a degenerate variant of the TEM-SGA, termed the TEM-SG, to facilitate further theoretical and numerical analysis. In this model, the network grows superlinearly by adding $t^{\theta }$ new nodes at each discrete time step *t* while setting λ=0 so that the edge selection probability reverts from Equation (3) to Equation (1). Each new node selects *m* existing edges and connects to both endpoints of each selected edge, thereby forming *m* new triangles upon attachment. By removing the aging mechanism, this design isolates the influence of triangle-based preferential attachment under superlinear growth, thus providing a tractable baseline for understanding the dominant role of triadic closure in network evolution (see Figure 9).

### 4.1. Degree Distribution P(k)

#### 4.1.1. Network Growth and Average Degree

In the TEM-SG, the number of new nodes added at each time *t* is $t^{\theta }$. We adopt a continuum approximation valid for large *t*, so sums over events are approximated by integrals, and state variables are differentiable in *t*. The total number of nodes N(t) satisfies

(4) $$\frac{dN}{dt}=t^{\theta }\;\Rightarrow \;N\left(t\right)=\int_{0}^{t}\tau^{\theta }\;d\tau ={\left[\frac{\tau^{\theta +1}}{\theta +1}\right]}_{0}^{t}=\frac{t^{\theta +1}}{\theta +1}.$$

Each new node connects to both endpoints of *m* existing edges, which introduces 2m new edges per node. Hence, the total number of edges E(t) evolves as

(5) $$\frac{dE}{dt}=2m\;t^{\theta }\;\Rightarrow \;E\left(t\right)=\int_{0}^{t}2m\;\tau^{\theta }\;d\tau ={\left[\frac{2m\;\tau^{\theta +1}}{\theta +1}\right]}_{0}^{t}=\frac{2m}{\theta +1}\;t^{\theta +1}.$$

Therefore, the average degree is constant:

(6) $$\bar{k}\left(t\right)=\frac{2E(t)}{N(t)}=\frac{2\cdot \frac{2m}{\theta +1}\;t^{\theta +1}}{\frac{t^{\theta +1}}{\theta +1}}=4m.$$

This shows that, although the network grows superlinearly, the average degree remains constant.

##### Remark (Model Detail)

The expression above assumes that each newly arrived node always introduces exactly 2m new edges and *m* new triangles by connecting to the endpoints of *m* base edges. If some of the intended edge-based attachments cannot be realized due to structural constraints (such as overlapping endpoints or forbidden multi-edges), our model applies a fallback rule to complete the connection process. This ensures that the growth relation in the above equations remains valid while preserving the structural consistency of the model. Although the fallback may occasionally alter some local attachment choices, its occurrence becomes increasingly rare as the network expands. Therefore, the fallback does not affect the asymptotic scaling behavior, and the degree distribution still follows the same power-law exponents.

#### 4.1.2. Node Degree Dynamics

Let $k_{i}\left(t\right)$ be the degree of node *i* at time *t*, and let $t_{i}$ be its birth time. Define the triangle-weighted degree

(7) $$K_{i}\left(t\right)=\sum_{j\in N(i)}k_{ij}\left(t\right),$$

where $k_{ij}\left(t\right)$ is the number of triangles that include edge (i,j).

During an infinitesimal interval dt, there are $t^{\theta }dt$ new nodes, each creating *m* triangle-based connections. Thus, the total number of edge-selection events per unit time is $mt^{\theta }$. The probability that a particular edge (i,j) is chosen is $P_{ij}=\frac{k_{ij}\left(t\right)}{S(t)},$ where

(8) $$S\left(t\right)=\sum_{\left(u,v)\in E(t\right)}k_{uv}\left(t\right)$$

is the sum of triangle counts over all edges.

Every time edge (i,j) is selected, node *i*’s degree increases by 1, and its triangle-weighted degree $K_{i}$ increases by 2 (the base edge (i,j) gains one triangle, and the newly created edge incident to *i* has triangle count 1). Hence,

(9) $$\frac{dk_{i}}{dt}=\frac{mt^{\theta }}{S(t)}\;K_{i}\left(t\right),\;\frac{dK_{i}}{dt}=\frac{2mt^{\theta }}{S(t)}\;K_{i}\left(t\right).$$

Taking the ratio yields

(10) $$\frac{dk_{i}/dt}{dK_{i}/dt}=\frac{1}{2}\;\Rightarrow \;\frac{dk_{i}}{dt}=\frac{1}{2}\frac{dK_{i}}{dt}\;\Rightarrow \;k_{i}\left(t\right)=\frac{1}{2}K_{i}\left(t\right)+C.$$

Using the initial condition $k_{i}\left(t_{i}\right)=K_{i}\left(t_{i}\right)=2m$, we obtain C=m, and thus

(11) $$k_{i}\left(t\right)=\frac{1}{2}K_{i}\left(t\right)+m.$$

#### 4.1.3. Solving for S(t)

Let T(t) be the number of triangles. Each selection event creates exactly one new triangle, and there are $mt^{\theta }$ selections per unit time; hence,

(12) $$\frac{dT}{dt}=m\;t^{\theta }\;\Rightarrow \;T\left(t\right)=\frac{m}{\theta +1}\;t^{\theta +1}.$$

Each triangle contributes +1 to the triangle count of each of its three incident edges; therefore,

(13) $$S\left(t\right)=\sum_{e}k_{e}\left(t\right)=3\;T\left(t\right)=\frac{3m}{\theta +1}\;t^{\theta +1}.$$

A Useful Identity (Consistency)

By double counting,

(14) $$\sum_{i}K_{i}\left(t\right)=\sum_{i}\sum_{j\in N(i)}k_{ij}\left(t\right)=2\sum_{\left(i,j)\in E(t\right)}k_{ij}\left(t\right)=2\;S\left(t\right),$$

which is preserved by the dynamics above.

Substituting (13) into (9) for the $K_{i}$-equation yields

(15) $$\frac{dK_{i}}{dt}=\frac{2mt^{\theta }}{S(t)}\;K_{i}=\frac{2mt^{\theta }}{\frac{3m}{\theta +1}\;t^{\theta +1}}\;K_{i}=\frac{2(\theta +1)}{3}\cdot \frac{K_{i}}{t}.$$

Solve

(16) $$\frac{1}{K_{i}}\frac{dK_{i}}{dt}=\frac{2(\theta +1)}{3t}\;\Rightarrow \;\int \frac{1}{K_{i}}\;dK_{i}=\int \frac{2(\theta +1)}{3t}\;dt\;\Rightarrow \;\ln K_{i}=\frac{2(\theta +1)}{3}\ln t+C_{1},$$

and

(17) $$K_{i}\left(t\right)=C_{2}\;t^{\frac{2(\theta +1)}{3}}.$$

Using $K_{i}\left(t_{i}\right)=2m$, we determine

(18) $$C_{2}=2m\cdot t_{i}^{-\frac{2(\theta +1)}{3}}\;\Rightarrow \;K_{i}\left(t\right)=2m{\left(\frac{t}{t_{i}}\right)}^{\frac{2(\theta +1)}{3}}.$$

Therefore, by (11),

(19) $$k_{i}\left(t\right)=\frac{1}{2}K_{i}\left(t\right)+m=m\left[{\left(\frac{t}{t_{i}}\right)}^{\frac{2(\theta +1)}{3}}+1\right],$$

and, for $t\gg t_{i}$,

(20) $$k_{i}\left(t\right)\approx m{\left(\frac{t}{t_{i}}\right)}^{\frac{2(\theta +1)}{3}}.$$

#### 4.1.4. Deriving the Degree Distribution

From (20),

(21) $$k_{i}\left(t\right)\approx m{\left(\frac{t}{t_{i}}\right)}^{\frac{2(\theta +1)}{3}}\;\Rightarrow \;t_{i}\approx t{\left(\frac{m}{k}\right)}^{\frac{3}{2(\theta +1)}}.$$

Assuming a continuous distribution of birth times among nodes present at time *t*, we have

(22) $$p\left(t_{i}\right)=\frac{dN/dt_{i}}{N(t)}=\frac{t_{i}^{\theta }}{\frac{t^{\theta +1}}{\theta +1}}=\frac{\left(\theta +1\right)\;t_{i}^{\theta }}{t^{\theta +1}}.$$

Let $t_{i}^{*}=t{\left(\frac{m}{k}\right)}^{\frac{3}{2(\theta +1)}}$. Then,

(23) $$P\;\left(k_{i}\left(t\right)<k\right)=P\;\left(t_{i}>t_{i}^{*}\right)=1-\int_{0}^{t_{i}^{*}}p\left(t_{i}\right)\;dt_{i}=1-{\left(\frac{m}{k}\right)}^{\frac{3}{2}}.$$

Differentiating provides the density

(24) $$P\left(k\right)=\frac{d}{dk}P\;\left(k_{i}\left(t\right)<k\right)=\frac{3}{2}\;m^{3/2}\;k^{-5/2},$$

which indicates a power-law tail with exponent γ=2.5 independent of θ.

### 4.2. Generalized Degree Distribution $P(k_{\ell })$

#### 4.2.1. Dynamics of Edge Triangle Count

Let $k_{e}\left(t\right)$ denote the number of triangles that edge *e* is part of at time *t*. Every new node joins by selecting *m* existing edges; at time *t*, the number of arrivals per unit time is $t^{\theta }$; hence, the total number of edge-selection events per unit time is

(25) $$m\;t^{\theta }.$$

Conditioned on existence of edge *e* at time *t*, the probability that *e* is selected in one event is

(26) $$P_{e}=\frac{k_{e}\left(t\right)}{S(t)},$$

where $S\left(t\right)=\sum_{e^{\prime }\in E\left(t\right)}k_{e^{\prime }}\left(t\right)$ is as in (8). Therefore,

(27) $$\frac{dk_{e}}{dt}=mt^{\theta }\cdot \frac{k_{e}\left(t\right)}{S(t)}.$$

From (13),

(28) $$\frac{dk_{e}}{dt}=mt^{\theta }\cdot \frac{k_{e}\left(t\right)}{\frac{3m}{\theta +1}t^{\theta +1}}=\frac{\theta +1}{3}\;\frac{k_{e}\left(t\right)}{t}.$$

Solving the separable ODE,

(29) $$\frac{1}{k_{e}\left(t\right)}\frac{dk_{e}}{dt}=\frac{\theta +1}{3t}\;\Rightarrow \;\int \frac{1}{k_{e}\left(t\right)}\;dk_{e}\left(t\right)=\int \frac{\theta +1}{3t}\;dt,$$

we obtain

(30) $$\ln k_{e}\left(t\right)=\frac{\theta +1}{3}\ln t+C_{1}\;\Rightarrow \;k_{e}\left(t\right)=C_{2}\;t^{\frac{\theta +1}{3}}.$$

Let $t_{e}$ be the birth time of edge *e*. By construction, $k_{e}\left(t_{e}\right)=1$, so

(31) $$1=C_{2}\cdot t_{e}^{\frac{\theta +1}{3}}\;\Rightarrow \;C_{2}=t_{e}^{-\frac{\theta +1}{3}},$$

and hence

(32) $$k_{e}\left(t\right)={\left(\frac{t}{t_{e}}\right)}^{\frac{\theta +1}{3}}.$$

#### 4.2.2. Birth Time Distribution of Edges

At each time *t*, there are $t^{\theta }$ new nodes, and each adds 2m new edges; thus,

(33) $$\frac{dE}{dt}=2m\;t^{\theta }.$$

Normalizing this rate over [0,t], the birth-time density of edges is

(34) $$p\left(t_{e}\right)=\frac{2m\cdot t_{e}^{\theta }}{\int_{0}^{t}2m\cdot \tau^{\theta }\;d\tau }=\frac{t_{e}^{\theta }}{\frac{t^{\theta +1}}{\theta +1}}=\frac{\left(\theta +1\right)\;t_{e}^{\theta }}{t^{\theta +1}}.$$

#### 4.2.3. Distribution of $k_{\ell }$

Let $k_{\ell }\equiv k_{e}\left(t\right)$. From (32),

(35) $$k_{e}\left(t\right)={\left(\frac{t}{t_{e}}\right)}^{\frac{\theta +1}{3}}\;\Rightarrow \;t_{e}=t\cdot k_{e}^{-\frac{3}{\theta +1}}.$$

Thus,

(36) $$P\;\left(k_{e}\left(t\right)<k_{\ell }\right)=P\;(t_{e}>t\cdot k_{\ell }^{-\frac{3}{\theta +1}}).$$

Using the cumulative distribution of $t_{e}$,

(37) $$P\left(t_{e}\leq \tau \right)=\int_{0}^{\tau }p\left(t_{e}\right)\;dt_{e}=\int_{0}^{\tau }\frac{\left(\theta +1\right)\;t_{e}^{\theta }}{t^{\theta +1}}\;dt_{e}={\left[\frac{t_{e}^{\theta +1}}{t^{\theta +1}}\right]}_{0}^{\tau }=\frac{\tau^{\theta +1}}{t^{\theta +1}},$$

and substituting $\tau =t\cdot k_{\ell }^{-\frac{3}{\theta +1}}$, we obtain

(38) $$P\;\left(k_{e}\left(t\right)<k_{\ell }\right)=1-\frac{{\left(t\cdot k_{\ell }^{-\frac{3}{\theta +1}}\right)}^{\theta +1}}{t^{\theta +1}}=1-k_{\ell }^{-3}.$$

Differentiating with respect to $k_{\ell }$ yields

(39) $$P\left(k_{\ell }\right)=\frac{d}{dk_{\ell }}\left[1-k_{\ell }^{-3}\right]=3\;k_{\ell }^{-4},$$

which reveals a power-law tail with exponent γ=4 independent of θ.

### 4.3. Verification of Theoretical Predictions

**Figure 10:**
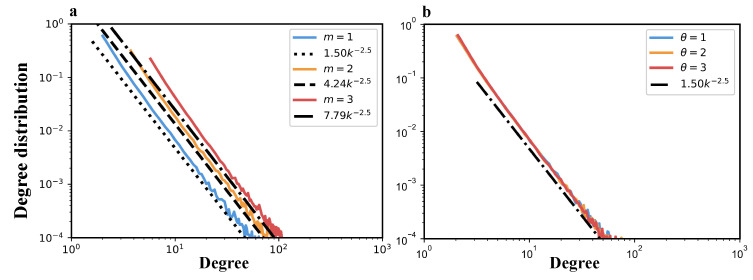
Topological properties of the TEM-SG. Log–log plots of degree distribution P(k) under various parameter settings are presented based on networks of size *N* = 10,000 generated using the TEM-SG across 100 realizations of the growth process (solid colored lines). Subfigure (**a**) shows the effect of varying the edge selection number *m* with fixed θ=1, while subfigure (**b**) illustrates the effect of varying the growth exponent θ with fixed m=1. The black dash-dotted lines represent the theoretical prediction $P\left(k\right)=\frac{3}{2}\;m^{3/2}\;k^{-5/2}$ derived in Equation (24).

**Figure 11:**
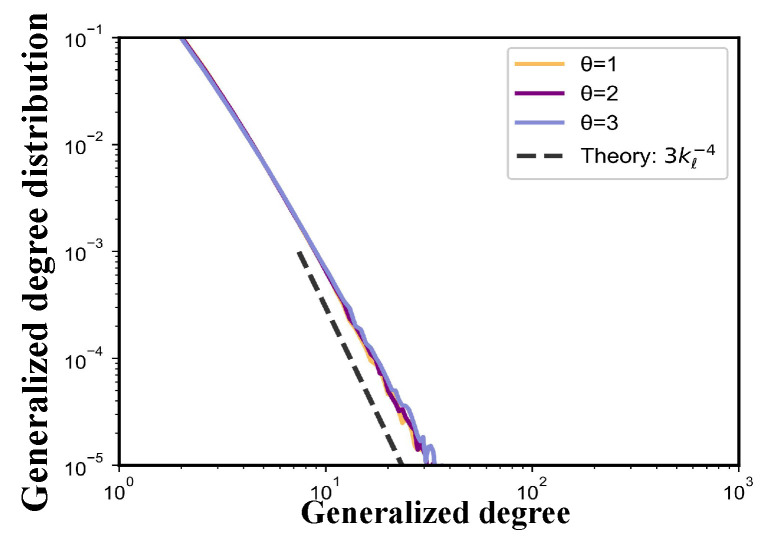
Topological properties of the TEM-SG. Log–log plots of the generalized degree distribution $P(k_{\ell })$ under various parameter settings are presented based on simulations of networks with N=10,000 nodes generated by the TEM-SG across 100 realizations of the growth process (shown as solid colored lines). Figure depicts the generalized degree distributions for variations in θ with fixed m=1. The dash-dotted lines indicate the theoretical scaling $P\left(k_{\ell }\right)=3k_{\ell }^{-4}$ predicted by Equation (39).

To validate the theoretical predictions derived in Section 4.1 and Section 4.2, we conduct numerical simulations of the TEM-SG under various parameter settings. In particular, we focus on examining whether the degree distribution and generalized degree distribution obtained from simulations align with the analytically derived power-law exponents. To this end, Figure 10 and Figure 11 are provided to visualize the alignment between theory and simulation.

Figure 10 shows the degree distributions of the TEM-SG, comparing theoretical predictions (dash-dotted lines) with simulation results (solid lines). In Figure 10a,b, the simulated distributions exhibit a clear power-law behavior with a slope of −2.5, consistent with the analytical prediction in Equation (24).

Notably, the theoretical expression $P\left(k\right)=\frac{3}{2}m^{3/2}k^{-2.5}$ shows that, although the power-law exponent remains constant, the parameter *m* induces a horizontal shift in the degree distribution. In Figure 10a, increasing *m* shifts the distribution curves upward, confirming that *m* controls the scale but not the exponent. In contrast, Figure 10b shows that varying the growth exponent θ does not affect the power-law exponent as the distribution curves for θ=1,2,3 substantially overlap across most of the degree range. These results validate the analytical framework developed in Section 4.1 and confirm that the TEM-SG accurately reproduces the theoretical degree distribution with exponent γ=2.5.

Figure 11 shows the generalized degree distributions $P(k_{\ell })$ of the TEM-SG, comparing theoretical predictions (dash-dotted lines) with simulation results (solid colored lines). In Figure 11, the simulated distributions exhibit a clear power-law decay with a slope of −4, in agreement with the analytical expression $P\left(k_{\ell }\right)=3k_{\ell }^{-4}$ derived in Equation (39).

Same as the node degree distribution, variations in θ have minimal impact on the overall shape and scaling behavior of the generalized degree distribution, indicating the robustness of the predicted power-law exponent to changes in θ.

Taken together, the results presented in Figure 10 and Figure 11 demonstrate consistency between simulation outcomes and analytical predictions for both the node degree and generalized degree distributions. In both cases, the distributions exhibit clear power-law behavior, with exponents precisely matching those derived theoretically—$P\left(k\right)∼k^{-2.5}$ and $P\left(k_{\ell }\right)∼k_{\ell }^{-4}$, respectively. This close agreement underscores the validity of the continuum approximation and the mean-field assumptions employed in the derivations [38,39], and it affirms the theoretical soundness of the TEM-SG across both node- and edge-level structural characterizations.

## 5. Conclusions

In this study, we introduced the Triangular Evolutionary Model of Superlinear Growth and Aging (TEM-SGA), an improved generative model for the evolution of citation networks. Through extensive numerical simulations, we demonstrated that the TEM-SGA is able to reproduce several empirical features of real citation systems, including degree distributions, generalized degree distributions, and local clustering patterns. We further conducted a series of experiments to investigate how the generated network topology varies under different parameter settings, and to examine the interplay between superlinear growth and node aging. To disentangle the contributions of different mechanisms, we also examined a degenerate variant, the TEM-SG, which excludes node aging. In this simplified setting, we derived theoretical expressions for both degree and generalized degree distributions, thereby providing deeper insight into the roles of superlinear growth and triadic closure in shaping network structure, and we conducted experiments to validate the correctness of these theoretical results.

### 5.1. Limitations

Despite these contributions, several limitations of the present work should be acknowledged. First, the model has been primarily validated on citation datasets, and its generalizability to other types of complex networks such as collaboration or social networks remains to be tested. Second, the current model assumes a unified superlinear growth pattern throughout the entire network evolution. However, our empirical findings indicate that superlinear dynamics predominantly characterize the post-1995 period, whereas earlier stages of citation growth are better captured by linear or exponential trends. This simplification limits the applicability of the model to the full temporal range of real-world citation networks. Third, while the fallback rule guarantees continuous evolution under structural constraints, it may not fully capture the diversity of linking strategies observed in practice. Finally, our theoretical analysis has mainly focused on the degenerate TEM-SG without aging as rigorous treatment of the full TEM-SGA remains mathematically challenging.

### 5.2. Future Directions

Addressing the aforementioned limitations opens several promising avenues for future research. A rigorous theoretical treatment of the complete TEM-SGA, despite its complexity, could provide deeper insights into the interplay between superlinear growth, node aging, and triadic closure. Extensions of the model could also explore hybrid evolutionary mechanisms that combine direct node-based attachment with the triangular closure mechanism studied here, thereby better reflecting the complexity of real citation practices. Moreover, future models could incorporate piecewise or time-varying growth regimes to account for the heterogeneous growth dynamics observed across different historical phases. In particular, the integration of linear, exponential, and superlinear components could enhance the realism of network growth modeling over long timescales. Finally, incorporating higher-order frameworks such as simplicial complexes or hypergraphs [40,41,42] would allow the model to capture group-level citation patterns beyond pairwise interactions. Recent studies suggest that higher-order growth processes can generate rich topological features and power-law behaviors across multiple scales, highlighting the potential of such approaches for modeling scholarly communication.

## Figures and Tables

**Table 1:** Symbols and parameter descriptions.

Symbol	Description
$N_{0}$	Number of nodes in the initial fully connected graph.
*N*	Total number of nodes when the network evolution ends.
*t*	Discrete time step.
θ	Growth exponent in the node-arrival process $t^{\theta }$.
λ	Aging rate controlling the decay of node attractiveness.
*m*	Number of base edges selected by each new node.
$a_{i}$	Attractiveness of node $v_{i}$ at time *t*.
N(t)	Number of nodes present in the network at time step *t*.
E(t)	the edge set at time *t* when used inside sums ( $\sum_{\left(i,j)\in E(t\right)}\;\cdot$); the number of edges |E(t)| when used as a scalar in growth/ODE expressions.
S(t)	Edge triangle mass
T(t)	Number of triangles at time *t* (so that S(t)=3T(t)).
E(t)	Explicit notation for the edge set at time *t*
N(i)	Set of neighboring nodes of node *i*.
$K_{i}\left(t\right)$	Triangle-based weight incident to node *i*
$k_{i}\left(t\right)$	Degree of node $v_{i}$ at time *t*.
$k_{ij}\left(t\right)$	Number of triangles that include edge (i,j) at time *t*.
$\bar{k}\left(t\right)$	Average degree of the network at time step *t*.
$P_{ij}\left(t\right)$	Probability that edge (i,j) is selected as a base edge by a new node at time *t*.

**Table 2:** Segment-wise fit comparison on log-scale. Lower log–MSE is better; higher $R^{2}$ is better. Best per segment is in bold.

Segment	Baseline	Log–MSE	$R^{2}$
1930–1952	Exponential	0.095	0.570
	**Linear**	**0.057**	**0.744**
	Superlinear	2.997	−12.521
1952–1995	**Exponential**	**0.076**	**0.871**
	Linear	3.723	−5.316
	Superlinear	1.072	−0.818
1995–2020	Exponential	0.737	−7.992
	Linear	10.800	−130.846
	**Superlinear**	**0.038**	**0.533**

**Table 3:** Structural comparison of average clustering coefficient (ACC) across the DBLP citation network, the proposed TEM-SGA, and classical models, including Barabási–Albert (BA), Holme–Kim (HK) [28], Klemm–Eguíluz Deactivation (KE-D) [37], and Li-Chen model (LC) [16], with N=10,000 nodes.

Model	m (Nodes/Edges)	θ	λ	ACC
DBLP (Dataset)	-	-	-	0.1200
BA	3	-	-	0.0058
HK (p=0.2)	3	-	-	0.1051
HK (p=0.7)	3	-	-	0.3852
KE-D (a=2)	3	-	-	0.7668
KE-D (a=5)	3	-	-	0.7366
LC (M=1000)	3	-	-	0.0079
LC (M=3000)	5	-	-	0.0067
TEM-SGA	3	2.0	0.0	0.2039
	3	1.0	0.0	0.1831
	3	4.0	0.0	0.2735
	4	2.0	0.0	0.1595
	3	2.0	0.2	0.1581
	3	2.8	0.1	0.1849

## Data Availability

The data is sourced from the following public websites: https://www.kaggle.com/datasets/mathurinache/citation-network-dataset, accessed on 23 December 2024 and https://networkrepository.com/cit-DBLP.php, accessed on 15 April 2025.

## Abbreviations

The following abbreviations are used in this manuscript:

TEM-SGA	Triangular Evolutionary Model of Superlinear Growth and Aging
TEM-SG	Triangular Evolutionary Model of Superlinear Growth
ACC	Average Clustering Coefficient
BA	Barabási–Albert Model
HK	Holme–Kim Model
KE-D	Klemm–Eguíluz Deactivation Model
LC	Li–Chen Model
